# Tests of a Two-Photon Technique for Measuring Polarization Mode Dispersion With Subfemtosecond Precision

**DOI:** 10.6028/jres.104.001

**Published:** 1999-02-01

**Authors:** Eric Dauler, Gregg Jaeger, Antoine Muller, A. Migdall, A. Sergienko

**Affiliations:** National Institute of Standards and Technology, Gaithersburg, MD 20899-0001; Dept. of Electrical and Computer Engineering, Boston University, 44 Cummington St., Boston, MA 02215

**Keywords:** coincidence, entangled photons, parametric downconversion, polarization interferometer, polarization mode dispersion (PMD), quantum interference

## Abstract

An investigation is made of a recently introduced quantum interferometric method capable of measuring polarization mode dispersion (PMD) on sub-femtosecond scales, without the usual interferometric stability problems associated with such small time scales. The technique makes use of the extreme temporal correlation of orthogonally polarized pairs of photons produced via type-II phase-matched spontaneous parametric down-conversion. When sent into a simple polarization interferometer these photon pairs produce a sharp interference feature seen in the coincidence rate. The PMD of a given sample is determined from the shift of that interference feature as the sample is inserted into the system. The stability and resolution of this technique is shown to be below 0.2 fs. We explore how this precision is improved by reducing the length of the down-conversion crystal and increasing the spectral band pass of the system.

## 1. Introduction

The highly correlated nature of photons produced two at a time via parametric down-conversion has proved to be an extremely useful tool in exploring the strange nature of quantum mechanics. In particular, type-II parametric down-conversion, which can produce photon pairs entangled in both space-time and polarization, has provided a wealth of interesting new interferometric phenomena [1–8]. One outgrowth of these studies is an application that allows the difference in propagation times between two polarization modes (otherwise known as polarization mode dispersion or PMD) to be determined with sub-femtosecond resolution. It is the extreme constraint on the simultaneity of the creation of the two photons of a pair that allows for the high resolution of the method. We explore the operating parameters of the method and their effect on the ultimate resolutions achievable.

The method has a number of distinct differences with respect to conventional PMD measurement methods that may be exploited to advantage. A high stability is seen without taking any of the special precautions usually required by conventional interferometric optical measurement systems (such as white light interferometer systems). This stability is due to the common path design of this simple two-photon interferometer configuration. Another advantage of the method (relative to some nonwhite light interferometric methods) is that it determines the optical delay absolutely, as opposed to simply measuring the delay modulo the wavelength.

## 2. Measurement Principle

Parametric down-conversion is a nonlinear process that takes place in an optically nonlinear crystal that allows an individual pump photon to, in effect, decay into a pair of photons. Because this decay occurs under the constraints of energy and momentum conservation, or phase matching, and because photons of a pair must be created virtually simultaneously, they are highly correlated. Our application uses a type-II phase matching arrangement, where a pump photon with extraordinary polarization is converted into a photon pair consisting of one extraordinary (e) polarization and one ordinary (o) polarization photon. Specifically, in the application here the down-conversion process is arranged so that the pair of output photons are emitted collinearly. This collinear pair of correlated photons enters a simple interferometer yielding an interference feature whose position is sensitive to the delay of one photon of a pair relative to the other.^2^ The PMD of a sample is directly determined from the shift of the interference feature produced by the insertion of a sample into the interferometer.

**Fig. 1:**
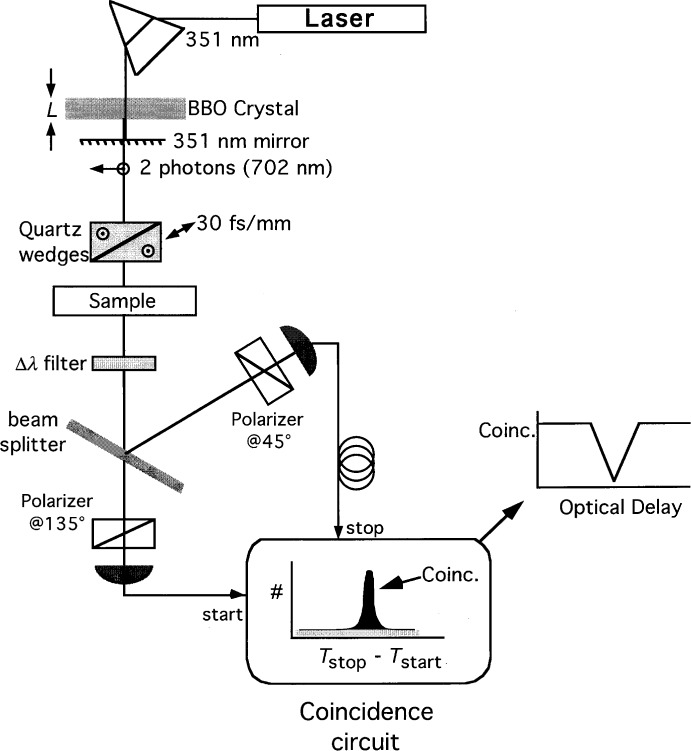
PMD measurement scheme.

The interferometer consists of a single beamsplitter, a pair of polarizers, and a pair of detectors (see Fig. 1). The collinear photon pairs encounter a polarization-insensitive 50-50 beamsplitter followed by one detector to catch the transmitted photons and one detector to catch the reflected photons. Down-converted pairs are registered as coincidences between the two detectors. This optical arrangement allows for two ways of producing a coincidence. The e-photon could be detected at detector 1 and the o-photon detected by detector 2, or vice versa. When these two ways of producing a coincidence event are arranged so that they cannot be distinguished (even in principle), quantum interference may be seen.

It is useful to describe here how coincidences are actually observed. Coincidence measurement systems typically use one detector to start a clock and a second detector to stop it. A fixed delay is added to the second detection channel to ensure that the stop pulse occurs after the start. A histogram of these start-stop time intervals is recorded. Correlated photon pairs are seen as a peak in pair detections at some specific delay between start and stop (see Fig. 1 inset). This is seen on top of a flat background due to uncorrelated single-detection firings of the two detectors.

There are two requirements for achieving indistinguishability and observing the quantum interference. First, polarizers must be placed before each of the detectors and oriented at 458 to the polarization of the e and o down-converted photons. These polarizers destroy the information as to whether each individual detected photon was an e-ray or o-ray, a requirement for indistinguishability. The second constraint arises because the e-ray and o-ray photons travel at different speeds through the optical system components (specifically, the down-conversion crystal and the sample under test). This results in an arrival time difference that also could, in principle, be used to determine which polarization photon was registered at each detector, thus destroying the indistinguishability of the two types of coincidence events. This time difference would be seen in the coincidence system as two separate coincidence peaks (at different time delays between start and stop detections), one due to each coincidence type which we refer to as 1e2o and 1o2e. (Note that the width of these correlated peaks is ultimately limited by the correlation time of the down-converted photons, assuming no electronic limit to the timing circuits.) Indistinguishability can be restored by adjusting the delay of the two photons in the common optical path so that the peaks due to both types of coincidence overlap. In this way, one can no longer tell (even in principle) whether a particular coincidence is an 1e2o or 1o2e type, allowing the two to interfere. In the present configuration, this indistinguishability condition is met when the two photons reach the beamsplitter simultaneously to within their coherence times. (This should not be interpreted to suggest that any interaction occurs between the photons on the beamsplitter, as it is possible to use other arrangements to achieve indistinguishability without having the two photons at the beamsplitter at the same time; see Ref. 9).

A differential delay line is used to delay one polarization relative to the other. The coincidence rate is recorded as this delay line is varied. When the two photons are separated at the beamsplitter by more than their coherence time, the two coincidence events can be distinguished, so no interference is possible and the total coincidence rate is just the sum of the two individual rates. When the two photons reach the beamsplitter to within their coherence time, then destructive or constructive interference can occur, depending on whether the detector polarizers are oriented at 45°–45° or 45°–135°. The inset of Fig. 1 shows the destructive interference signature in the coincidence signal as the delay between the two photons is varied.

The following is an intuitive explanation of the interference profile (for a more rigorous derivation, see the appendices). The width of this interference dip is mainly due to birefringence of the down-conversion crystal itself and any bandwidth limiting elements. The dip has a finite width because, although the two photons of a pair are created simultaneously (or nearly so), the relative delays encountered after creation are not necessarily identical for all pairs. First, photon pairs created at different points within the crystal traverse different lengths of the crystal before exiting, which, because the crystal is birefringent, leads to a range of relative delays for the emitted photon pairs. From this it is easy to see that shorter crystals yield a narrower interference feature, the width being on the order of (*n*_e_–*n*_o_)*L*/*c*, where *L* is the crystal length, *n*_e_ and *n*_o_ are the e and o indices of refraction, and *c* is the speed of light. A spectrum limiting element can add to the spread of relative delays by adding random delays to individual photons. (This occurs in an interference filter because it operates as a resonant cavity where photons “rattle back and forth” with some probability for exiting on each bounce.) Thus, reducing the spectral bandpass of the system beyond a certain point broadens the observed dip.

The shape of the interference dip is a convolution of the temporal correlation or coherence functions of the down-converted light. When temporal correlations are limited by the length of the down-conversion crystal, a triangular shaped interference feature is seen. This occurs because the two wavefunctions convolved are rectangular in shape. At the other extreme, when the coherence time of a spectral filter dominates the system, the shape approaches a Gaussian because the temporal coherence of a spectral filter is typically Gaussian [8]. In general, the final shape will be intermediate between a triangular shape and a Gaussian shape. A derivation of these shapes is given in the appendices.

The PMD of a sample is found by scanning the differential delay to find the center of the interference dip, both with the sample inserted into and removed from the optical path. The shift of the center of the interference feature is the PMD of the sample. The uncertainty limit of the method is determined by how well the center of that feature can be found. The main thrust of this paper is to explore how crystal length and spectral passband affect the measurement uncertainty.

## 3. Experiment

As shown in Fig. 1, a 351 nm, 0.5 W laser was used to pump a *β* -BaB_2_O_4_ (BBO) crystal oriented to produce orthogonally polarized, but collinearly propagating, down-conversion photons at a center frequency of 702 nm. A polarization insensitive 50-50 beam splitter directed the down-converted photons to two polarizers oriented at 45° to the e- and o-ray photons and at 0° to each other. A prism before the BBO crystal was used to reject laser light other than the 351 nm beam. A high efficiency 351 nm mirror after the BBO blocked the pump light from the system, while passing the longer wavelength down-converted light. The polarization delay line, essentially a continuously variable thickness birefringent plate, was made from a pair of identical 15 mm by 15 mm by 30 mm quartz wedges (with their optic axes oriented out of the page in the perspective of Fig. 1). One wedge was fixed while the other could be translated along its hypotenuse. The differential delay produced by this variable thickness quartz plate of 30 fs/mm was determined from published index of refraction data [10]. Bandpass filters of various widths centered at 702 nm were placed in the common path just before the beamsplitter. (The wavelengths, 351 nm and 702 nm, used in this experiment were chosen simply for ease of laser light generation and optical detection. It is certainly of interest to move these measurements to the communication wavelengths, as photon counting detectors become more readily available.) For a PMD measurement, a sample would be placed after the wedges, although the tests described here only determine how well the center of the interference feature can be determined, which is crucial to find the ultimate limit of PMD measurements that could be made using this arrangement.

## 4. Results

Several data series were taken. The interference dip was mapped as a function of both spectral band limiting and BBO crystal length. In addition, a time series of repeated scans over a dip was taken for each crystal length to determine the resolution and stability of the measurement system. This last set of data gives a feel for the ultimate PMD measurement capability of this technique.

**Fig. 2:**
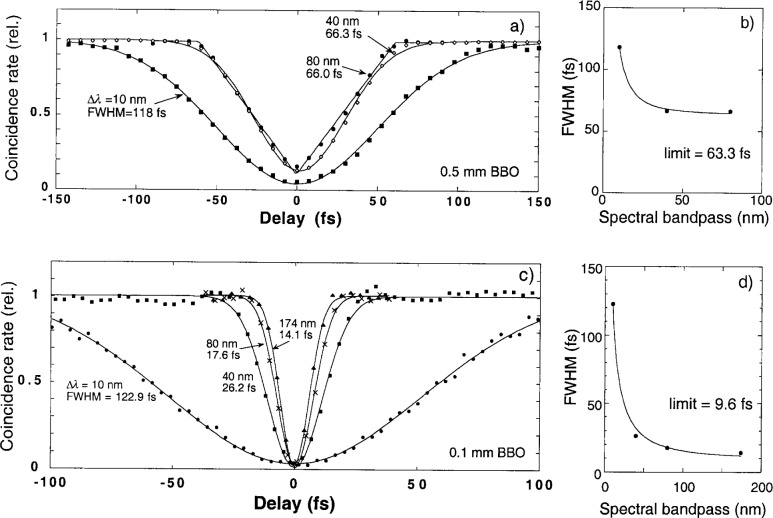
a) Interference profile of 0.5 mm long BBO crystal for three spectral filters. b) Full width at half maximum (FWHM) of the interference dip versus spectral filter bandpass. c) Interference profile of 0.1 mm long BBO crystal for four spectral filters. d) FWHM of the interference dip versus spectral filter bandpass.

Figure 2a shows how the interference profile made using a 0.5 mm BBO varies as different spectral filters are installed. The dip width is seen to narrow as the spectral passband is increased. As the dip width decreases, its shape changes from Gaussian to triangular, indicating that the coherence time of the two-photons changes from being limited by the spectral filter to being limited by the crystal length. Figure 2b shows the full width half at maximum (FWHM) of the dip approaching a constant as the bandpass is increased. The points were fit to a function of the form $FHWM=\sqrt{a^{2}+(\frac{b}{\Delta \lambda }{)}^{2}}$, which assumes that the total width is the quadrature sum of two components, one a constant (*a*) due to the crystal length and the other inversely proportional to the spectral filter. The fitted value for *a* was 63.3 fs. The parameter *b*, giving the proportionality between the inverse bandpass and the coherence time, can be calculated for various passband shapes. Typically the value can be expected to fall between 1 and 0.32, the calculated values for rectangular and Lorentzian passbands respectively (a Gaussian shape yields 0.66 [11]). In this case (Fig. 2b), the fitted value of *b* was 0.62, which is within the expected range, especially considering that only three data points were taken. For this data set, it is clear that we are able to reach the regime where the dip profile is limited by the crystal length.

Figure 2c shows the dip profile made using a 0.1 mm BBO crystal, again with a range of spectral filters. As before, the profile width narrows as the spectral band is increased, although even at the largest bandpass of 174 nm we cannot say that we have definitively made the transition to the triangular profile shape indicative of a crystal length limited coherence time. While Fig. 2c is not as definitive as the 0.5 mm BBO measurements, Fig. 2d is, showing that further increase of the bandpass will not significantly reduce the dip width. (The fitted value for *b* here is 0.76, again within the expected range.) The fact that a triangular shape was not reached here is likely due in part to the subtle transition between the two shapes (see the 80 nm and 40 nm shapes in Fig. 2a). In fact, the 174 nm scan can be fit about as well with a Gaussian shape or triangular shape.

**Fig. 3:**
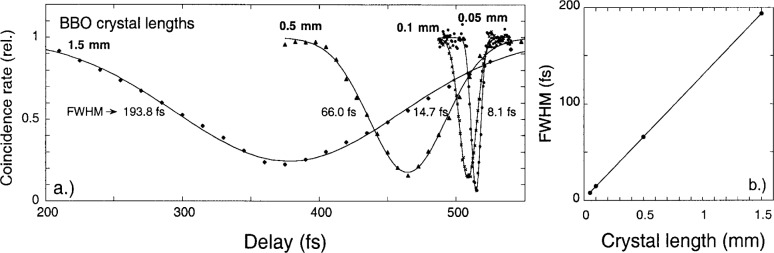
a) Interference profile for four BBO crystal lengths. b) Dip FWHm versus BBO length.

Interference dips were measured for four different thickness BBO crystals with broad spectral filters so as not to limit the width of the interference dip (see Fig. 3a). Table 1 lists, for each of these measurements, the spectral bandwidths of the filters used and their associated coherence times, as well as the correlation time of the down-converted light due solely to crystal length. As shown in the table, the crystal correlation time exceeds the filter coherence time for all the measurements except the one made with the 0.05 mm BBO. In that instance there may have been some broadening of the dip due to a too-narrow spectral filter, although as mentioned in the table caption the actual filter coherence time is somewhat smaller than the 9.4 fs value in the table calculated for a rectangular shape. (A wider filter was not used here because of excessive detector count rates. Also, the fact that the longest crystals did not provide sharp triangular interference dips may be due to optical misalignments and will be investigated further, although this deviation from the ideal situation did not significantly affect the final results here.) The linearity of the FWHM data shown in Fig. 3b provides further support that each of these measurements is mainly limited by the crystal length rather than the spectral filter width.

**Fig. 4:**
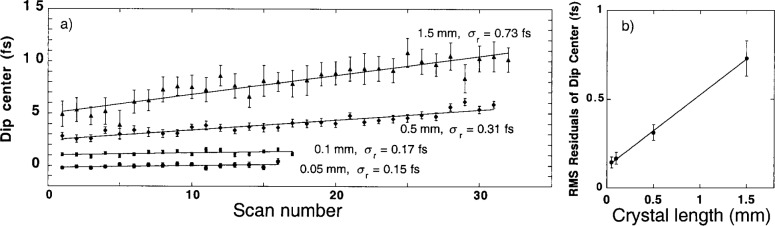
a) Drift of the interference dip center with successive scans for four BBO crystal lengths. An arbitrary shift was introduced between the data sets for clarity of the figure. The data were fit to a line for each crystal and a residual scatter about that line determined (sr is the rms of the residuals, that is $\sigma =\sqrt{{\left(x_{i}-f_{i}\right)}^{2}}/N$, where *x*_i_ is the center of an individual scan i, *f*_i_ is the fit value of that scan, and *N* is the number of scans). b) The residual scatter versus crystal length.

Measurements of temporal variations of the interference dip centers were used as an indicator of the ultimate stability and resolution of the PMD method. Figure 4a shows the variation over successive scans of the center of the interference dip. The error bars are the uncertainty of the fit parameter determination. The series for each of the four BBO crystal lengths exhibits a linear drift that decreases as the length decreases, which seems to indicate that the drift is associated with the crystal rather than any other component of the system. This may be due to temperature drift of the crystal, which was not thermally stabilized for these measurements. Figure 4b shows the residual scatter ***σ***_r_, of the data after removing the linear drift. This is an indication of the resolution and noise of the PMD measurement technique. This level of scatter is consistent with the uncertainty of the individual points. The resulting uncertainty limit of this method appears to be about 0.15 fs.

Exceeding this limit using the current measurement system may be difficult for several reasons. First, it is impractical to use a crystal much thinner than 0.05 mm, because the two-photon signal decreases linearly with crystal length, while ordinary one-photon surface scatter remains constant. In addition, it is difficult to fabricate crystals much smaller that this. The use of thinner crystals also requires wider spectral passbands. Because the 0.05 mm BBO crystal measurement already required a passband of 174 nm, it is hard to imagine that more than a factor of two improvement could be gained here. Wider spectral passbands introduce an additional practical problem: they allow more stray light to be seen by the detectors, increasing the background of accidental coincidences. Finally, Fig. 4b shows an intercept of about 0.15 fs, indicating no improvement even at zero crystal length. The origin of this nonzero intercept is not understood at this point and warrants further investigation.

**Fig. 5:**
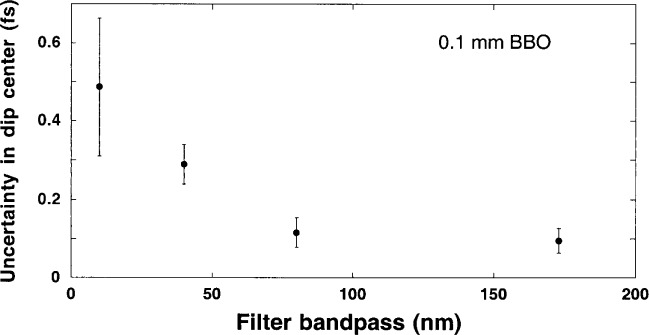
Uncertainty in locating the interference dip center for 0.1 mm long BBO as the spectral width was varied.

An additional analysis of measurement uncertainty was made using the data series of Fig. 2c, where the effect of bandwidth on the uncertainty of the dip center was explored. For a range of spectral widths (from (**Δ*λ*** = 10 to **Δ*λ*** = 174 nm), it was observed that restricting the spectral range also increased the uncertainty of optical delay determination (see Fig. 5). This agrees with the intuitive expectation that a dip broadened for whatever reason increases the uncertainty of its center location.

## 5. Conclusions

In conclusion, we have demonstrated a technique for measuring PMD with uncertainties as low as 0.15 fs. These results show that a simple two-photon interferometer with a common path geometry can be extremely stable, even without the usual engineering required for interferometric stability. It will be interesting to see what uncertainties can be achieved with proper attention to mechanical and thermal stability. We have shown that the best uncertainty is achieved with short crystal length and wide spectral bandpass and that practical systems can be made using crystals as thin as 0.05 mm.

Finally, new entirely solid state systems to produce entangled pairs of photons have already been constructed [12]. These convenient compact sources greatly advance the potential to turn this demonstration into a practical system for PMD measurement.

## Figures and Tables

**Fig. A1:**
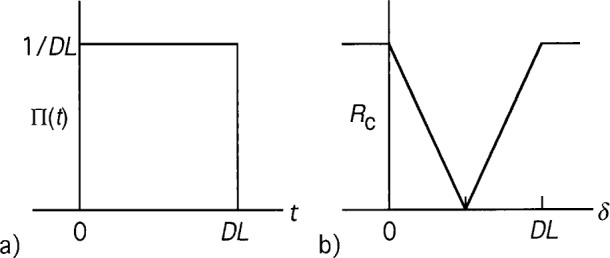
Two-photon wavefunction and coincidence profiles.

**Fig. C1:**
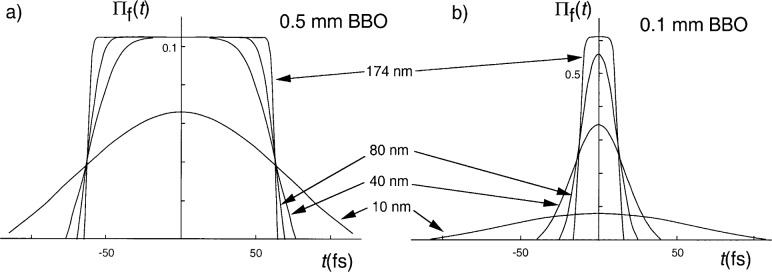
Two-photon wavefunction factors Π_f_(*t*) for *L* = 0.5 mm (a) and 0.1 mm (b) BBO crystals restricted by filter passbands of 10 nm, 40 nm, 80 nm, and 174 nm.

**Table 1:** Correlation times for system components

BBO length (mm)	1.5	0.5	0.1	0.05
Filter bandpass (nm)	40	80	174	174
Filter coherence time^a^ (fs) (Δ*t~* 1*/*Δ*v*)	41	20	9.4	9.4
BBO correlation time (fs) (*L*(*n*_e_–*n*_o_)/2*c*)	185	62	12	6.2
Observed FWHM (fs)	193.8	66.0	14.7	8.0

a The relation between bandpass and coherence time assumes a rectangular spectral bandpass shape. For the actual shape the correlation time would likely be reduced by about 10 % to 25 % [11].

## Footnotes

2 It is important to note that while much of the descriptive language here discusses the down-converted light as if it consists of pairs of two individual photons, the entanglement of the wavefunction is such that ultimately the down-converted light must be considered to inherently consist of single two-photons rather than pairs of ordinary single photons [9]. While this simplification is useful for an intuitive explanation of the measurement, it should be remembered that it cannot be carried too far without running into inconsistencies.

## 6. Appendix A. Two-Photon Wavefunction (Without Spectral Limiting)

In our experiment, a laser-pumped optically nonlinear crystal produces, through phase-matched type-II spontaneous parametric down-conversion, collinear frequency-degenerate photon pairs of orthogonal polarization. All pairs have a photon in each of the two polarization basis states, |o⟩ and |e⟩, corresponding to the ordinary and extraordinary rays of the (birefringent) down-converting crystal, respectively. These pairs are produced in a superposition of two polarization anti-correlated states, |o⟩|e⟩ and |e⟩|o⟩.

The state vector of the collinear pair of photons as they leave the nonlinear crystal is

$$\begin{array}{c} |\Psi \rangle =\int d\omega_{1}d\omega_{2}\delta \left(\omega_{1}-\omega_{2}-\omega_{p}\right)\\ \times \Psi \left(k_{1}+k_{2}-k_{p}\right)\;a_{o}^{†}\left(\omega_{1}\left(k_{1}\right)\right)\;a_{e}^{†}\left(\omega_{2}\left(k_{2}\right)\right)|0\rangle , \end{array}$$ (A1)

where *ω_i_* and *k_i_* (*i* = 1,2,p) represent the frequency and wave number (for the signal, idler and pump photons), respectively, and the *a_i_*^†^ are photon creation operators [8]. In Eq. (A1), the delta function enforces the frequency phase-matching condition of parametric down-conversion and the function *Ψ* (**Δ***k*) = [1 – exp(−i**Δ***kL*)]/(i**Δ***kL*) is the natural spectral distribution of the two-photon, where *L* is the crystal length and **Δ***k* = *k*_1_ + *k*_2_ − *k*_p_. The wave-number phase-matching condition is *k*_1_ + *k*_2_ = *k*_p_ (or **Δ***k* = 0). State (1) describes the doubly entangled two-photons of type-II down-conversion: for each pair *ω*_1_, *ω*_2_ of possible frequencies there is a two-photon superposition of the form

$$\left[a_{o}^{†}\left(\omega_{1}\right)\;a_{e}^{†}\left(\omega_{2}\right)+a_{e}^{†}\left(\omega_{1}\right)\;a_{o}^{†}\left(\omega_{2}\right)\right]|0\rangle .$$ (A2)

The down-converting crystal introduces a discrete optical delay (= *DL*/2) between e-polarized and o-polarized photons, and the beamsplitter is carefully aligned to match the polarization planes of the down-converting crystal, where $D=\frac{1}{V_{o}\left(\mathrm{BBO}\right)}-\frac{1}{V_{e}\left(\mathrm{BBO}\right)}$ with *V*_o_(BBO) and *V*_e_(BBO) being the ordinary and extraordinary group velocities. A birefringent sample (of length *l*) to be measured is positioned after the down-converting crystal. This introduces another discrete contribution to the optical delay *δ*, where *δ* = [*n*o (sample)−*n*_e_(sample)]*l*/*c*, with *n*_e_(sample) and *n*_o_(sample) referring to the extraordinary and ordinary indices of refraction of the sample.

For positive uniaxial crystals like quartz, *δ* is opposite in sign to the delay (*DL*/2) within a negative uniaxial crystal like BBO; the two discrete contributions work against one another when similarly oriented, i.e., there is optical delay compensation. Without the sample placed in the apparatus the profiles of the initial wave-function and count rate are as shown in Fig. A1 (see Appendix B).

After the sample is in place, instead of the initial wavefunction [cf. Eq. (B4)], one has

$$\begin{array}{l} \Psi \left(t_{1},t_{2}\right)=\alpha_{t}\alpha_{r}v\left(t_{1}+t_{2}+\phi \right)\;\left[u\left(t_{1}-t_{2}+\delta \right)-u\left(-t_{1}+t_{2}+\delta \right)\right], \end{array}$$ (A3)

where *ϕ* is a phase constant related to the sum of the two path lengths. The two terms in Eq. (A3) (each including a rectangular factor shown in Fig. A1a) can overlap and will cancel each other when |*δ*| = |*DL*/2|. The counting rate *R*_c_ [see Eq. (B3) and Fig. A1b] becomes

$$R_{c}=R_{0}\left[1-\rho \left(\delta \right)\right],$$ (A4)

where ***ρ*** (*δ*) is a Λ-shaped function with a half-base of *DL*/2. *R*_c_ is thus a constant with a V-shaped dip:

$$\rho =\{\begin{array}{cc} 0 & -\infty <\delta <0\\ \kappa \delta & 0<\delta <DL/2\\ 1-\kappa \left(\delta -DL/2\right) & DL/2\leq \delta <DL\\ 0 & DL<\delta <\infty , \end{array}$$ (A5)

where in turn ***κ*** ≡ 2/*DL*. It is the shift in the interference dip that is used to measure the optical delay of the sample.

## 7. Appendix B. Coincidence Rate

The electric fields after the polarizers, *E*_1_^(+)^ and *E*_2_^(+)^, are given by

$${E_{1}}^{\left(+\right)}(t)=\alpha_{t}\int d\omega \;g\left(\omega \right)\;\left[\exp\left(-i\omega {t_{1}}^{o}\right){\hat{e}}_{1}\cdot {\hat{e}}_{o}\;a_{o}\left(\omega \right)+\exp\left(-\omega {t_{1}}^{e}\right){\hat{e}}_{1}\cdot {\hat{e}}_{e}\;a_{e}\left(\omega \right)\right]$$ (B1)

$${E_{2}}^{\left(+\right)}(t)=\alpha_{r}\int d\omega \;g\left(\omega \right)\;\left[\exp\left(-i\omega {t_{2}}^{o}\right){\hat{e}}_{2}\cdot {\hat{e}}_{o}\;a_{o}\left(\omega \right)+\exp\left(-i\omega {t_{2}}^{e}\right){\hat{e}}_{2}\cdot {\hat{e}}_{e}\;a_{e}\left(\omega \right)\right],$$ (B2)

where ***ê****_i_* is in the direction of the *i* th linear polarizer axis (*i* = 1, 2) or photon polarization direction (*i* = o, e), *a*_o_(*ω*) and *a*_e_(*ω*) are annihilation operators for the o- and e-polarization photons, *α*_t_ and *α*_r_ are the complex transmission and reflection coefficients of the beamsplitter and *g*(*ω*) is the spectral transmission function of the bandpass filter. For large bandpasses, *g*(*ω*) is effectively one; for restricted bandpasses it is Gaussian. The times associated with the quantum amplitudes are given by $t_{i}^{j}\equiv t-s_{i}^{j}/c\;\left(i=1,2;j=o,e\right)$, where $s_{i}^{j}$ is the total optical path for the photons.

A coincidence anticorrelation arises from destructive quantum interference between two of the resulting quantum states: one state with an o-polarization photon going to detector 1 and an e-polarization photon going to detector 2, and another state with a e-polarization photon going to detector 1 and a o-polarization photon going to detector 2. Coincidences of two photons at a single detector also occur but are not monitored. The coincidence counting rate between the two detectors is given by

$$\begin{array}{l} R_{c}=\left(1/T\right)\int_{0}^{T}\int_{0}^{T}dT_{1}dT_{2}\langle \Psi |{E_{1}}^{\left(-\right)}{E_{2}}^{\left(-\right)}{E_{2}}^{\left(+\right)}{E_{1}}^{\left(+\right)}|\Psi \rangle \\ \;=\left(1/T\right)\int_{0}^{T}\int_{0}^{T}dT_{1}dT_{2}{\left|\Psi \left(t_{1},t_{2}\right)\right|}^{2} \end{array}$$ B3)

where *T_i_* (*i* = 1, 2) is the detection time of the *i* th detector, *T* is the time window of the coincidence circuit and

$$\Psi \left(t_{1},t_{2}\right)\;=\langle 0|{E_{1}}^{\left(+\right)}{E_{2}}^{\left(+\right)}|\Psi \rangle =\alpha_{t}\alpha_{r}v\left(t_{1}+t_{2}\right)\;\left[u\left(t_{1}-t_{2}\right)-u\left(-t_{1}+t_{2}\right)\right],$$ (B4)

where

$$\begin{array}{c} v\left(t\right)=v_{o}\exp\left(-i\omega_{p}t/2\right),\\ u\left(t\right)=u_{o}\exp\left(-i\omega_{d}t/2\right)\times \\ \left\{\int_{-\infty }^{\infty }dv\left[1-\exp\left(-vDL\right)\right]/\left(ivDL\right)\right\}\exp\left(-ivt\right)\\ =\exp\left(-i\omega_{d}t/2\right)\Pi \left(t\right), \end{array}$$ (B5)

with

$$\Pi \left(t\right)=\{\begin{array}{cc} u_{o} & DL>t>0\\ 0 & \text{otherwise}, \end{array}$$

where *u*_0_ and *v*_0_ are normalization constants and *ω*_d_ ≡ *Ω*_o_ − *Ω*_e_, with *Ω*_o_, *Ω*_e_ being the frequencies of the ordinary and extraordinary rays for perfect phase matching. The first term of Eq. (B4) describes the case of the o-polarization photon going to detector 1 and the e-polarization photon going to detector 2; the second term describes the opposite. The sign difference between terms occurs due to reflection at the beamsplitter.

## 8. Appendix C. Two-Photon Wave-function (With Spectral Limiting)

The form of the two-photon wavefunction incorporating a limited spectral bandwidth is determined by the factor

$$\Pi_{f}\left(t\right)=f_{0}\left\{\text{erf}\left(\sigma_{0}t/2\right)-\text{erf}\left[\left(\sigma_{0}t-DL\right)/2\right]\right\}/2DL.$$ (C1)

This function peaks at *t* = *DL*/2 and has a width on the order of *DL* + 8/*σ*_0_, where *σ*_0_ is the bandwidth in angular frequency [8]. Profiles for this function were calculated numerically for different crystal lengths and pass-bands (see Fig. C1).

The profile of Π_f_(*t*) varies from the rectangular shape of Π(*t*) for large bandpass ranges to a nearly Gaussian shape for small bandpass ranges. Accordingly, *R*_c_[cf. Eq. (B3)] has a V-shaped dip in the former range and a Gaussian dip in the latter.
